# Tris(2-acetyl­cyclo­pentan-1-onato-κ^2^ *O*,*O*′)aluminium

**DOI:** 10.1107/S1600536812021848

**Published:** 2012-05-19

**Authors:** Franc Perdih

**Affiliations:** a Faculty of Chemistry and Chemical Technology, University of Ljubljana, Aškerčeva 5, P. O. Box 537, SI-1000 Ljubljana, Slovenia, and CO EN–FIST, Dunajska 156, SI-1000 Ljubljana, Slovenia

## Abstract

In the title compound, [Al(C_7_H_9_O_2_)_3_], the Al^III^ cation is coordinated by six O atoms from three 2-acetyl­cyclo­penta­nonate ligands in a slightly distorted octa­hedral environment, with Al—O bond lengths in the range 1.882 (2)–1.896 (2) Å. In the crystal, mol­ecules are linked together *via* C—H⋯O inter­actions. One of the C atoms in one ring has a large thermal motion compared to the other atoms, indicating some possible disorder. However, the treatment of this C atom as disordered over two positions did not give a significant improvement.

## Related literature

For applications of metal complexes with β-diketones, see: Bray *et al.* (2007 ▶); Garibay *et al.* (2009 ▶); Lutz *et al.* (1989 ▶); Perdih (2011 ▶); Vreshch *et al.* (2004 ▶); Wu & Wang (2009 ▶). For related structures, see: Hon & Pfluger (1973 ▶); Schröder *et al.* (2011 ▶).
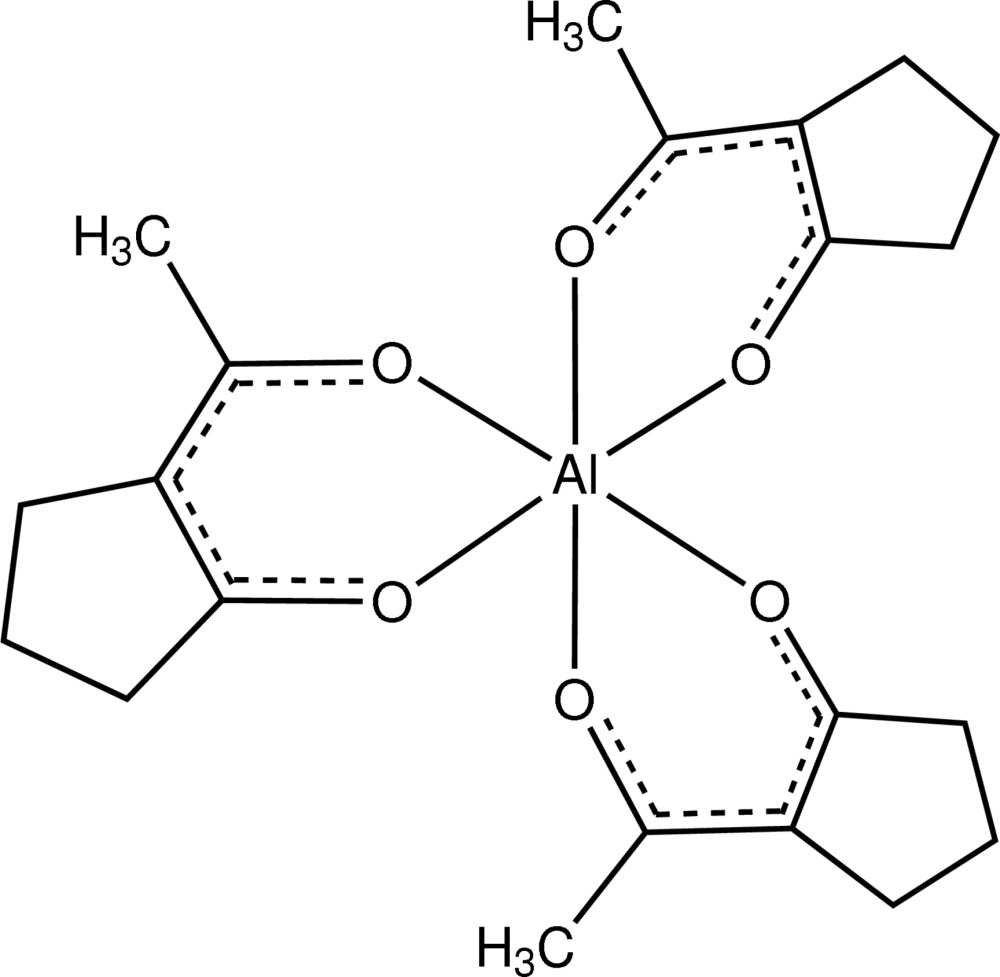


## Experimental

### 

#### Crystal data

[Al(C_7_H_9_O_2_)_3_]
*M* *_r_* = 402.41
Monoclinic, 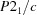

*a* = 8.1785 (3) Å
*b* = 15.7494 (6) Å
*c* = 15.6615 (5) Å
β = 94.039 (2)°
*V* = 2012.29 (12) Å^3^
*Z* = 4
Mo *K*α radiation
μ = 0.14 mm^−1^
*T* = 293 K
0.25 × 0.15 × 0.08 mm

#### Data collection

Nonius KappaCCD area-detector diffractometer
Absorption correction: multi-scan (*SCALEPACK*; Otwinowski & Minor, 1997 ▶) *T*_min_ = 0.967, *T*_max_ = 0.989
8554 measured reflections
4523 independent reflections
3007 reflections with *I* > 2σ(*I*)
*R*_int_ = 0.031

#### Refinement

*R*[*F* ^2^ > 2σ(*F* ^2^)] = 0.072
*wR*(*F* ^2^) = 0.228
*S* = 1.03
4523 reflections
256 parameters
H-atom parameters constrained
Δρ_max_ = 0.56 e Å^−3^
Δρ_min_ = −0.27 e Å^−3^

### 

Data collection: *COLLECT* (Nonius, 1998 ▶); cell refinement: *DENZO-SMN* (Otwinowski & Minor, 1997 ▶); data reduction: *DENZO-SMN*; program(s) used to solve structure: *SIR97* (Altomare *et al.*, 1999 ▶); program(s) used to refine structure: *SHELXL97* (Sheldrick, 2008 ▶); molecular graphics: *ORTEP-3 for Windows* (Farrugia, 1997 ▶) and *DIAMOND* (Brandenburg, 1999 ▶); software used to prepare material for publication: *WinGX* (Farrugia, 1999 ▶) and *publCIF* (Westrip, 2010 ▶).

## Supplementary Material

Crystal structure: contains datablock(s) I, global. DOI: 10.1107/S1600536812021848/fj2551sup1.cif

Structure factors: contains datablock(s) I. DOI: 10.1107/S1600536812021848/fj2551Isup2.hkl

Additional supplementary materials:  crystallographic information; 3D view; checkCIF report

## Figures and Tables

**Table 1:** Hydrogen-bond geometry (Å, °)

*D*—H⋯*A*	*D*—H	H⋯*A*	*D*⋯*A*	*D*—H⋯*A*
C12—H12*B*⋯O6^i^	0.97	2.54	3.427 (4)	153

Symmetry code: (i) 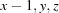.

**Fig. 1.**
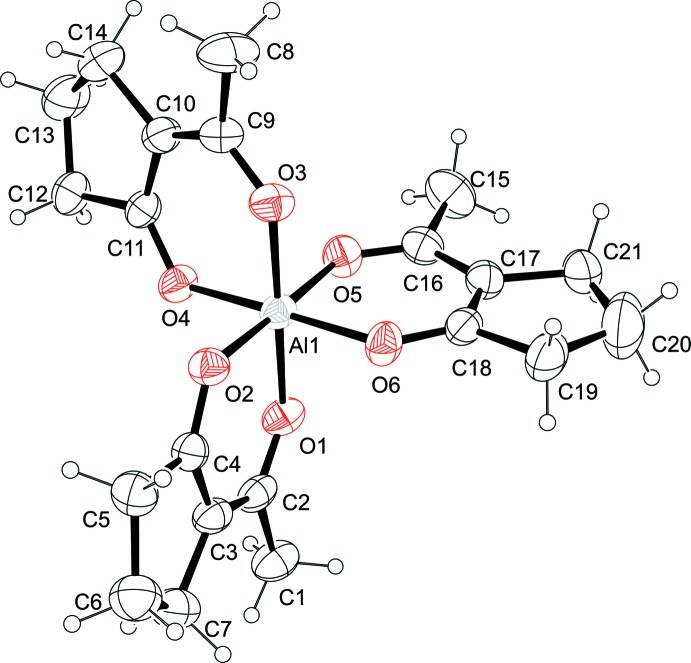
The molecular structure of the title complex showing the numbering scheme and displacement ellipsoids drawn at the 30% probability level.

**Fig. 2.**
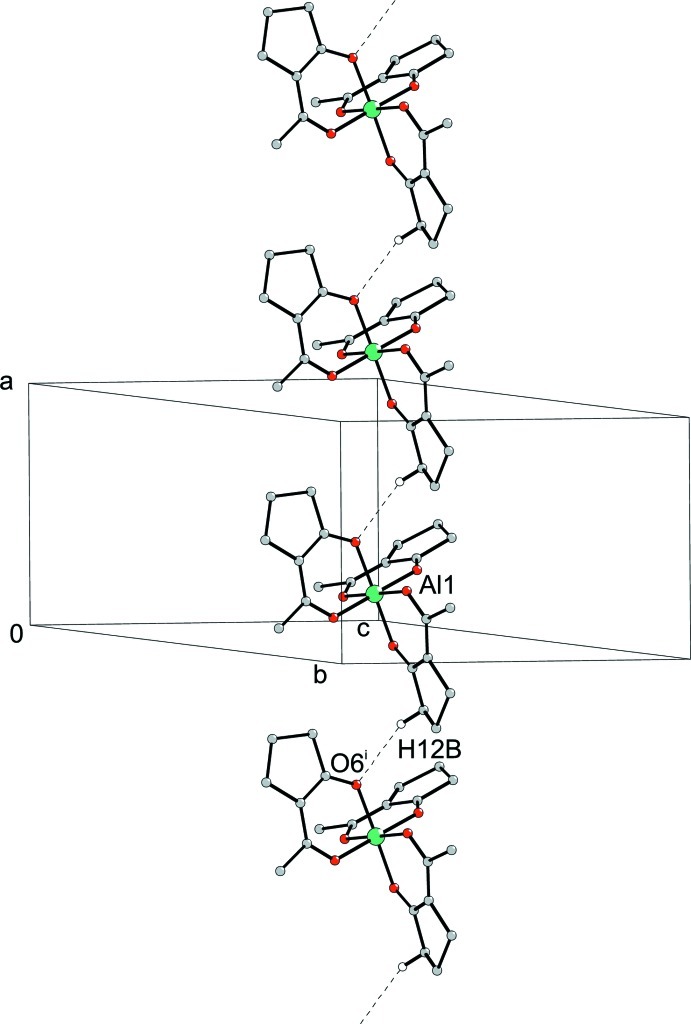
A chain formation. Dashed lines indicate intermolecular C12—H12B···O6 hydrogen bonding. For the sake of clarity, H atoms not involved in the motif shown have been omitted. Symmetry code: i = x – 1, y, z.

## supplementary crystallographic information

### Comment

β-Diketonates have been proven to be versatile ligands for various metal ions. They can be easily derivatized, thus modifying the electronic and steric nature of these ligands to design suitable structure/function relationship (Bray *et al.*, 2007; Garibay *et al.*, 2009; Perdih, 2011). β-diketonate compounds of aluminium have received great attention due to the promise of the construction of cages (Vreshch *et al.*, 2004; Wu & Wang, 2009). Besides that, the title compound is a close analogue of aluminium isomaltolato compound that was prepared for *in vivo* examinations of ion transport in order to shed some light on the mechanism of transport and the involvement of Al in neurological disorders (Lutz *et al.*, 1989).

In the title molecule (Fig. 1), the aluminium(III) cation is surrounded by six O atoms from three 2-acetylcyclopentanonate ligands in a octahedral environment. The geometry around aluminium is close to the orthogonallity as can be seen from the angles. The Al—O bond lengths in the range 1.882 (2)–1.896 (2) Å and are similar as for example in Al(acac)_3_ (Hon & Pfluger, 1973). All three cyclopentyl rings deviates from the expected envelope conformation and they are close to planarity, with torsion angles C3–C4–C5–C6 - 7.2 (4), C10–C11–C12–C13 - 17.6 (4)° and C17–C18–C19–C20 5.0 (5)°. Such small torsion angles are often observed in cyclopentyl and 1,3-dioxolyl rings condensed to aromatic or delocalized systems. This values are somewhat smaller then in analogues iron(III) compound (Schröder *et al.*, 2011). 1-D framework is achieved due to weak intermolecular C12–H12B···O6(*x* – 1, *y*, *z*) interactions (Fig. 2).

### Experimental

To a clear solution of Al_2_(SO_4_)_3_^.^18H_2_O (1 mmol, 0.67 g) in water (15 ml) a solution of 2-acetylcyclopentanone (6 mmol, 0.76 g) in methanol (5 ml) was added while stirring. Afterwards the 1 *M* NaOH (6 ml) was slowly added and the resulting solution stirred at 70°C for 15 minutes. After cooling to the room temperature the light pink product was filtrated and washed with water (20 ml), and subsequently air-dried. Yield: 0.47 g, 58%. Crystals suitable for X-ray analysis were obtained by recrystallization from ethanol.

### Refinement

All H atoms were initially located in a difference Fourier maps and were subsequently treated as riding atoms in geometrically idealized positions, with C–H = 0.97 (methylene) or 0.96 Å (methyl) and with *U*_iso_(H) = *kU*_eq_(C), where *k* = 1.5 for methyl groups, which were permitted to rotate but not to tilt, and 1.2 for all other H atoms. To improve the refinement results, one reflection with too high value of *δ*(*F*^2^)/e.s.d. and with *F*_o_^2^ < *F*_c_^2^ was deleted from the refinement. Displacement ellipsoid of C20 is large compared to the other atoms, however the treatement of C20 as disorderd over two positions did not improve the model.

### Crystal data

**Table:**

[Al(C_7_H_9_O_2_)_3_]	*F*(000) = 856
*M**_r_* = 402.41	*D*_x_ = 1.328 Mg m^−^^3^
Monoclinic, *P*2_1_/*c*	Mo *K*α radiation, λ = 0.71073 Å
Hall symbol: -P 2ybc	Cell parameters from 4677 reflections
*a* = 8.1785 (3) Å	θ = 2.6–27.5°
*b* = 15.7494 (6) Å	µ = 0.14 mm^−^^1^
*c* = 15.6615 (5) Å	*T* = 293 K
β = 94.039 (2)°	Prism, pink
*V* = 2012.29 (12) Å^3^	0.25 × 0.15 × 0.08 mm
*Z* = 4	

### Data collection

**Table:**

Nonius KappaCCD area-detector diffractometer	4523 independent reflections
Graphite monochromator	3007 reflections with *I* > 2σ(*I*)
Detector resolution: 0.055 pixels mm^-1^	*R*_int_ = 0.031
ω scans	θ_max_ = 27.4°, θ_min_ = 5.4°
Absorption correction: multi-scan (*SCALEPACK*; Otwinowski & Minor, 1997)	*h* = −10→10
*T*_min_ = 0.967, *T*_max_ = 0.989	*k* = −19→20
8554 measured reflections	*l* = −20→20

### Refinement

**Table:**

Refinement on *F*^2^	Primary atom site location: structure-invariant direct methods
Least-squares matrix: full	Secondary atom site location: difference Fourier map
*R*[*F*^2^ > 2σ(*F*^2^)] = 0.072	Hydrogen site location: inferred from neighbouring sites
*wR*(*F*^2^) = 0.228	H-atom parameters constrained
*S* = 1.03	*w* = 1/[σ^2^(*F*_o_^2^) + (0.1175*P*)^2^ + 1.0495*P*] where *P* = (*F*_o_^2^ + 2*F*_c_^2^)/3
4523 reflections	(Δ/σ)_max_ < 0.001
256 parameters	Δρ_max_ = 0.56 e Å^−^^3^
0 restraints	Δρ_min_ = −0.27 e Å^−^^3^

### Special details

**Table:**

Experimental. 259 frames in 6 sets of ω scans. Rotation/frame = 1.6 °. Crystal-detector distance = 25.00 mm. Measuring time = 135 s/°.
Geometry. All s.u.'s (except the s.u. in the dihedral angle between two l.s. planes) are estimated using the full covariance matrix. The cell s.u.'s are taken into account individually in the estimation of s.u.'s in distances, angles and torsion angles; correlations between s.u.'s in cell parameters are only used when they are defined by crystal symmetry. An approximate (isotropic) treatment of cell s.u.'s is used for estimating s.u.'s involving l.s. planes.
Refinement. Refinement of *F*^2^ against ALL reflections. The weighted *R*-factor *wR* and goodness of fit *S* are based on *F*^2^, conventional *R*-factors *R* are based on *F*, with *F* set to zero for negative *F*^2^. The threshold expression of *F*^2^ > 2σ(*F*^2^) is used only for calculating *R*-factors(gt) *etc*. and is not relevant to the choice of reflections for refinement. *R*-factors based on *F*^2^ are statistically about twice as large as those based on *F*, and *R*- factors based on ALL data will be even larger.

### Fractional atomic coordinates and isotropic or equivalent isotropic displacement parameters (Å^2^)

**Table:**

	*x*	*y*	*z*	*U*_iso_*/*U*_eq_	
Al1	0.14039 (11)	0.17092 (6)	0.83613 (6)	0.0532 (3)	
O1	0.1108 (3)	0.05260 (14)	0.85125 (16)	0.0650 (6)	
O2	0.2377 (3)	0.18225 (13)	0.94807 (14)	0.0597 (5)	
O3	0.1711 (3)	0.28903 (15)	0.82273 (15)	0.0652 (6)	
O4	−0.0712 (3)	0.18733 (13)	0.87403 (15)	0.0614 (6)	
O5	0.0428 (3)	0.16358 (16)	0.72368 (15)	0.0680 (6)	
O6	0.3511 (3)	0.15136 (16)	0.79798 (14)	0.0643 (6)	
C1	0.1271 (5)	−0.0881 (2)	0.9056 (3)	0.0782 (11)	
H1A	0.0111	−0.0969	0.8962	0.117*	
H1B	0.1669	−0.1168	0.957	0.117*	
H1C	0.1812	−0.1102	0.8579	0.117*	
C2	0.1616 (4)	0.0054 (2)	0.9145 (2)	0.0615 (8)	
C3	0.2455 (4)	0.0367 (2)	0.9884 (2)	0.0592 (8)	
C4	0.2763 (4)	0.1222 (2)	1.0002 (2)	0.0558 (7)	
C5	0.3627 (5)	0.1393 (3)	1.0861 (2)	0.0723 (9)	
H5A	0.292	0.1706	1.1221	0.087*	
H5B	0.4615	0.1722	1.08	0.087*	
C6	0.4041 (6)	0.0539 (3)	1.1243 (3)	0.0898 (12)	
H6A	0.3712	0.051	1.1825	0.108*	
H6B	0.5212	0.0437	1.1251	0.108*	
C7	0.3097 (5)	−0.0125 (3)	1.0675 (3)	0.0827 (11)	
H7A	0.382	−0.0581	1.0521	0.099*	
H7B	0.22	−0.0366	1.0969	0.099*	
C8	0.1261 (6)	0.4374 (3)	0.8147 (4)	0.0965 (14)	
H8A	0.2406	0.4399	0.8328	0.145*	
H8B	0.0665	0.4755	0.8489	0.145*	
H8C	0.1105	0.4536	0.7556	0.145*	
C9	0.0646 (4)	0.3488 (2)	0.8254 (2)	0.0618 (8)	
C10	−0.0975 (4)	0.3343 (2)	0.8415 (2)	0.0613 (8)	
C11	−0.1526 (4)	0.25647 (19)	0.86725 (19)	0.0544 (7)	
C12	−0.3253 (4)	0.2623 (2)	0.8937 (3)	0.0708 (9)	
H12A	−0.3274	0.2635	0.9555	0.085*	
H12B	−0.3908	0.2149	0.8714	0.085*	
C13	−0.3866 (5)	0.3453 (3)	0.8545 (4)	0.0978 (14)	
H13A	−0.4617	0.3728	0.8909	0.117*	
H13B	−0.4427	0.3356	0.7987	0.117*	
C14	−0.2323 (5)	0.4006 (3)	0.8464 (3)	0.0885 (12)	
H14A	−0.2423	0.4352	0.7951	0.106*	
H14B	−0.212	0.4371	0.8959	0.106*	
C15	0.0005 (6)	0.1580 (4)	0.5720 (3)	0.1018 (15)	
H15A	−0.1099	0.1444	0.5841	0.153*	
H15B	0.0376	0.1184	0.5309	0.153*	
H15C	0.0041	0.2145	0.5492	0.153*	
C16	0.1110 (5)	0.1527 (2)	0.6540 (2)	0.0656 (8)	
C17	0.2757 (4)	0.1371 (2)	0.6491 (2)	0.0637 (8)	
C18	0.3859 (4)	0.1378 (2)	0.7208 (2)	0.0601 (8)	
C19	0.5569 (5)	0.1223 (3)	0.6982 (3)	0.0901 (13)	
H19A	0.605	0.0743	0.7296	0.108*	
H19B	0.625	0.172	0.7096	0.108*	
C20	0.5346 (8)	0.1031 (6)	0.5998 (4)	0.146 (3)	
H20A	0.6077	0.1387	0.5693	0.175*	
H20B	0.562	0.0442	0.5893	0.175*	
C21	0.3599 (6)	0.1200 (3)	0.5679 (2)	0.0795 (11)	
H21A	0.3123	0.0711	0.5378	0.095*	
H21B	0.3525	0.1688	0.5301	0.095*	

### Atomic displacement parameters (Å^2^)

**Table:**

	*U*^11^	*U*^22^	*U*^33^	*U*^12^	*U*^13^	*U*^23^
Al1	0.0445 (5)	0.0518 (5)	0.0634 (6)	0.0021 (4)	0.0050 (4)	0.0008 (4)
O1	0.0590 (13)	0.0509 (12)	0.0843 (15)	0.0013 (10)	−0.0007 (11)	−0.0063 (11)
O2	0.0647 (13)	0.0506 (11)	0.0634 (12)	−0.0003 (10)	0.0028 (10)	0.0003 (10)
O3	0.0507 (12)	0.0577 (13)	0.0871 (16)	−0.0048 (10)	0.0049 (11)	0.0115 (11)
O4	0.0499 (12)	0.0530 (12)	0.0826 (15)	−0.0004 (9)	0.0134 (10)	0.0020 (11)
O5	0.0501 (12)	0.0859 (17)	0.0672 (14)	0.0022 (11)	−0.0006 (10)	0.0017 (12)
O6	0.0453 (11)	0.0830 (16)	0.0647 (13)	0.0085 (10)	0.0057 (9)	−0.0047 (11)
C1	0.070 (2)	0.0508 (18)	0.114 (3)	−0.0067 (16)	0.007 (2)	−0.0023 (19)
C2	0.0438 (15)	0.0476 (16)	0.095 (2)	0.0028 (12)	0.0168 (15)	0.0003 (16)
C3	0.0484 (16)	0.0518 (16)	0.078 (2)	0.0044 (13)	0.0108 (14)	0.0075 (15)
C4	0.0490 (16)	0.0591 (17)	0.0602 (17)	0.0036 (13)	0.0098 (13)	0.0031 (14)
C5	0.072 (2)	0.077 (2)	0.067 (2)	−0.0015 (18)	−0.0044 (17)	0.0000 (17)
C6	0.093 (3)	0.088 (3)	0.088 (3)	0.010 (2)	−0.005 (2)	0.006 (2)
C7	0.069 (2)	0.079 (2)	0.101 (3)	0.0039 (19)	0.009 (2)	0.026 (2)
C8	0.084 (3)	0.058 (2)	0.146 (4)	−0.0124 (19)	−0.004 (3)	0.022 (2)
C9	0.0634 (19)	0.0520 (16)	0.0686 (19)	−0.0011 (15)	−0.0040 (15)	0.0059 (14)
C10	0.0619 (19)	0.0563 (17)	0.0654 (18)	0.0077 (15)	0.0026 (15)	0.0013 (15)
C11	0.0469 (15)	0.0561 (17)	0.0598 (17)	0.0030 (13)	0.0016 (12)	−0.0059 (13)
C12	0.0502 (18)	0.080 (2)	0.083 (2)	0.0036 (16)	0.0112 (16)	−0.0066 (19)
C13	0.067 (2)	0.102 (3)	0.124 (4)	0.023 (2)	0.012 (2)	0.008 (3)
C14	0.087 (3)	0.074 (2)	0.105 (3)	0.028 (2)	0.010 (2)	0.005 (2)
C15	0.104 (4)	0.122 (4)	0.077 (3)	−0.016 (3)	−0.013 (2)	0.010 (3)
C16	0.069 (2)	0.0590 (18)	0.068 (2)	−0.0097 (16)	0.0007 (16)	0.0029 (15)
C17	0.071 (2)	0.0559 (17)	0.0649 (19)	−0.0040 (16)	0.0126 (16)	0.0000 (15)
C18	0.0561 (17)	0.0505 (16)	0.075 (2)	0.0022 (13)	0.0158 (15)	0.0019 (15)
C19	0.071 (2)	0.089 (3)	0.114 (3)	0.012 (2)	0.030 (2)	0.000 (2)
C20	0.108 (4)	0.223 (8)	0.111 (4)	0.045 (5)	0.040 (3)	0.004 (5)
C21	0.097 (3)	0.080 (2)	0.064 (2)	0.004 (2)	0.0191 (19)	−0.0013 (18)

### Geometric parameters (Å, º)

**Table:**

Al1—O2	1.882 (2)	C8—H8B	0.96
Al1—O5	1.885 (2)	C8—H8C	0.96
Al1—O4	1.887 (2)	C9—C10	1.386 (5)
Al1—O6	1.889 (2)	C10—C11	1.377 (4)
Al1—O3	1.891 (2)	C10—C14	1.524 (5)
Al1—O1	1.896 (2)	C11—C12	1.502 (4)
O1—C2	1.284 (4)	C12—C13	1.514 (6)
O2—C4	1.274 (4)	C12—H12A	0.97
O3—C9	1.285 (4)	C12—H12B	0.97
O4—C11	1.277 (4)	C13—C14	1.545 (7)
O5—C16	1.272 (4)	C13—H13A	0.97
O6—C18	1.279 (4)	C13—H13B	0.97
C1—C2	1.504 (5)	C14—H14A	0.97
C1—H1A	0.96	C14—H14B	0.97
C1—H1B	0.96	C15—C16	1.519 (5)
C1—H1C	0.96	C15—H15A	0.96
C2—C3	1.392 (5)	C15—H15B	0.96
C3—C4	1.380 (4)	C15—H15C	0.96
C3—C7	1.524 (5)	C16—C17	1.376 (5)
C4—C5	1.499 (5)	C17—C18	1.389 (5)
C5—C6	1.501 (6)	C17—C21	1.512 (5)
C5—H5A	0.97	C18—C19	1.486 (5)
C5—H5B	0.97	C19—C20	1.569 (7)
C6—C7	1.544 (6)	C19—H19A	0.97
C6—H6A	0.97	C19—H19B	0.97
C6—H6B	0.97	C20—C21	1.504 (7)
C7—H7A	0.97	C20—H20A	0.97
C7—H7B	0.97	C20—H20B	0.97
C8—C9	1.496 (5)	C21—H21A	0.97
C8—H8A	0.96	C21—H21B	0.97
			
O2—Al1—O5	178.08 (11)	O3—C9—C10	123.0 (3)
O2—Al1—O4	91.78 (11)	O3—C9—C8	116.5 (3)
O5—Al1—O4	87.99 (11)	C10—C9—C8	120.4 (3)
O2—Al1—O6	88.54 (10)	C11—C10—C9	122.5 (3)
O5—Al1—O6	91.73 (10)	C11—C10—C14	110.0 (3)
O4—Al1—O6	178.47 (11)	C9—C10—C14	127.0 (3)
O2—Al1—O3	87.68 (10)	O4—C11—C10	127.2 (3)
O5—Al1—O3	90.43 (11)	O4—C11—C12	121.6 (3)
O4—Al1—O3	91.84 (10)	C10—C11—C12	111.1 (3)
O6—Al1—O3	89.67 (11)	C11—C12—C13	103.4 (3)
O2—Al1—O1	91.50 (10)	C11—C12—H12A	111.1
O5—Al1—O1	90.40 (11)	C13—C12—H12A	111.1
O4—Al1—O1	88.12 (10)	C11—C12—H12B	111.1
O6—Al1—O1	90.38 (11)	C13—C12—H12B	111.1
O3—Al1—O1	179.17 (12)	H12A—C12—H12B	109
C2—O1—Al1	128.9 (2)	C12—C13—C14	105.7 (3)
C4—O2—Al1	126.6 (2)	C12—C13—H13A	110.6
C9—O3—Al1	128.4 (2)	C14—C13—H13A	110.6
C11—O4—Al1	125.1 (2)	C12—C13—H13B	110.6
C16—O5—Al1	128.9 (2)	C14—C13—H13B	110.6
C18—O6—Al1	126.5 (2)	H13A—C13—H13B	108.7
C2—C1—H1A	109.5	C10—C14—C13	102.5 (3)
C2—C1—H1B	109.5	C10—C14—H14A	111.3
H1A—C1—H1B	109.5	C13—C14—H14A	111.3
C2—C1—H1C	109.5	C10—C14—H14B	111.3
H1A—C1—H1C	109.5	C13—C14—H14B	111.3
H1B—C1—H1C	109.5	H14A—C14—H14B	109.2
O1—C2—C3	123.4 (3)	C16—C15—H15A	109.5
O1—C2—C1	116.5 (3)	C16—C15—H15B	109.5
C3—C2—C1	120.1 (3)	H15A—C15—H15B	109.5
C4—C3—C2	122.1 (3)	C16—C15—H15C	109.5
C4—C3—C7	109.8 (3)	H15A—C15—H15C	109.5
C2—C3—C7	128.1 (3)	H15B—C15—H15C	109.5
O2—C4—C3	127.1 (3)	O5—C16—C17	124.2 (3)
O2—C4—C5	121.4 (3)	O5—C16—C15	116.5 (4)
C3—C4—C5	111.4 (3)	C17—C16—C15	119.3 (4)
C4—C5—C6	106.0 (3)	C16—C17—C18	122.4 (3)
C4—C5—H5A	110.5	C16—C17—C21	125.7 (3)
C6—C5—H5A	110.5	C18—C17—C21	111.9 (3)
C4—C5—H5B	110.5	O6—C18—C17	126.2 (3)
C6—C5—H5B	110.5	O6—C18—C19	121.8 (3)
H5A—C5—H5B	108.7	C17—C18—C19	112.0 (3)
C5—C6—C7	106.8 (3)	C18—C19—C20	102.7 (4)
C5—C6—H6A	110.4	C18—C19—H19A	111.2
C7—C6—H6A	110.4	C20—C19—H19A	111.2
C5—C6—H6B	110.4	C18—C19—H19B	111.2
C7—C6—H6B	110.4	C20—C19—H19B	111.2
H6A—C6—H6B	108.6	H19A—C19—H19B	109.1
C3—C7—C6	104.6 (3)	C21—C20—C19	109.5 (4)
C3—C7—H7A	110.8	C21—C20—H20A	109.8
C6—C7—H7A	110.8	C19—C20—H20A	109.8
C3—C7—H7B	110.8	C21—C20—H20B	109.8
C6—C7—H7B	110.8	C19—C20—H20B	109.8
H7A—C7—H7B	108.9	H20A—C20—H20B	108.2
C9—C8—H8A	109.5	C20—C21—C17	103.4 (4)
C9—C8—H8B	109.5	C20—C21—H21A	111.1
H8A—C8—H8B	109.5	C17—C21—H21A	111.1
C9—C8—H8C	109.5	C20—C21—H21B	111.1
H8A—C8—H8C	109.5	C17—C21—H21B	111.1
H8B—C8—H8C	109.5	H21A—C21—H21B	109.1
			
O2—Al1—O1—C2	−6.5 (3)	C4—C3—C7—C6	−7.2 (4)
O5—Al1—O1—C2	173.8 (3)	C2—C3—C7—C6	175.3 (3)
O4—Al1—O1—C2	−98.2 (3)	C5—C6—C7—C3	11.3 (4)
O6—Al1—O1—C2	82.0 (3)	Al1—O3—C9—C10	1.7 (5)
O4—Al1—O2—C4	95.2 (2)	Al1—O3—C9—C8	178.5 (3)
O6—Al1—O2—C4	−83.3 (3)	O3—C9—C10—C11	8.9 (5)
O3—Al1—O2—C4	−173.0 (3)	C8—C9—C10—C11	−167.7 (4)
O1—Al1—O2—C4	7.1 (3)	O3—C9—C10—C14	179.6 (4)
O2—Al1—O3—C9	−102.5 (3)	C8—C9—C10—C14	3.0 (6)
O5—Al1—O3—C9	77.2 (3)	Al1—O4—C11—C10	−8.6 (5)
O4—Al1—O3—C9	−10.8 (3)	Al1—O4—C11—C12	175.7 (2)
O6—Al1—O3—C9	169.0 (3)	C9—C10—C11—O4	−5.3 (6)
O2—Al1—O4—C11	101.5 (3)	C14—C10—C11—O4	−177.4 (3)
O5—Al1—O4—C11	−76.6 (3)	C9—C10—C11—C12	170.8 (3)
O3—Al1—O4—C11	13.7 (3)	C14—C10—C11—C12	−1.3 (4)
O1—Al1—O4—C11	−167.1 (3)	O4—C11—C12—C13	−166.0 (3)
O4—Al1—O5—C16	179.1 (3)	C10—C11—C12—C13	17.6 (4)
O6—Al1—O5—C16	−2.4 (3)	C11—C12—C13—C14	−26.3 (5)
O3—Al1—O5—C16	87.3 (3)	C11—C10—C14—C13	−15.1 (4)
O1—Al1—O5—C16	−92.8 (3)	C9—C10—C14—C13	173.2 (4)
O2—Al1—O6—C18	−179.3 (3)	C12—C13—C14—C10	25.4 (5)
O5—Al1—O6—C18	−1.2 (3)	Al1—O5—C16—C17	5.4 (5)
O3—Al1—O6—C18	−91.6 (3)	Al1—O5—C16—C15	−174.2 (3)
O1—Al1—O6—C18	89.2 (3)	O5—C16—C17—C18	−4.6 (5)
Al1—O1—C2—C3	3.3 (5)	C15—C16—C17—C18	175.0 (3)
Al1—O1—C2—C1	−176.4 (2)	O5—C16—C17—C21	177.1 (3)
O1—C2—C3—C4	1.9 (5)	C15—C16—C17—C21	−3.3 (6)
C1—C2—C3—C4	−178.4 (3)	Al1—O6—C18—C17	2.0 (5)
O1—C2—C3—C7	179.2 (3)	Al1—O6—C18—C19	−179.4 (3)
C1—C2—C3—C7	−1.1 (5)	C16—C17—C18—O6	0.8 (5)
Al1—O2—C4—C3	−4.8 (5)	C21—C17—C18—O6	179.3 (3)
Al1—O2—C4—C5	176.3 (2)	C16—C17—C18—C19	−177.9 (3)
C2—C3—C4—O2	−1.1 (5)	C21—C17—C18—C19	0.6 (4)
C7—C3—C4—O2	−178.9 (3)	O6—C18—C19—C20	176.1 (4)
C2—C3—C4—C5	177.9 (3)	C17—C18—C19—C20	−5.1 (5)
C7—C3—C4—C5	0.1 (4)	C18—C19—C20—C21	7.8 (6)
O2—C4—C5—C6	−173.7 (3)	C19—C20—C21—C17	−7.5 (6)
C3—C4—C5—C6	7.2 (4)	C16—C17—C21—C20	−177.1 (5)
C4—C5—C6—C7	−11.3 (4)	C18—C17—C21—C20	4.4 (5)

### Hydrogen-bond geometry (Å, º)

**Table:**

*D*—H···*A*	*D*—H	H···*A*	*D*···*A*	*D*—H···*A*
C12—H12*B*···O6^i^	0.97	2.54	3.427 (4)	153

Symmetry code: (i) *x*−1, *y*, *z*.
